# Infant feeding information, attitudes and practices: a longitudinal survey in central Nepal

**DOI:** 10.1186/1746-4358-9-14

**Published:** 2014-08-28

**Authors:** Rajendra Karkee, Andy H Lee, Vishnu Khanal, Colin W Binns

**Affiliations:** 1 School of Public Health and Community Medicine, BP Koirala Institute of Health Sciences, Dharan, Nepal; 2 School of Public Health, Curtin University, Perth, WA, Australia; 3 Sanjeevani College of Medical Sciences, Butwal, Rupandehi, Nepal

**Keywords:** Attitude, Breastfeeding, Feeding practice, Nepal

## Abstract

**Background:**

Infant feeding is governed by environmental as well as cultural factors. Breastfeeding knowledge and attitudes are known to be associated with breastfeeding duration. This study investigated breastfeeding information, attitudes and supplementary feeding in the central hills district of Nepal.

**Methods:**

A community-based prospective cohort study of 701 pregnant women was conducted. Information on breastfeeding attitudes, feeding practices and supplementary feeding was sought from the cohort at 4 weeks, 12 weeks and 22 weeks postpartum through repeated interviews using validated questionnaires.

**Results:**

Average duration of intended breastfeeding was 28 months (SD 7.9) and average target time to introduce solid foods was 6.1 months (SD 1.2). About 80% of women reported their husband, mother/mother-in-law preferred breastfeeding. Eleven percent of the cohort said that breastfeeding was not enjoyable. At 12 weeks and 22 weeks after birth, about a quarter (24.8%) and half (52.8%) of the infants were introduced cow/buffalo milk, respectively, while only 6.3% and 13.4% of them were given infant formula. Overall, any breastfeeding rate remained high at over 98% throughout the follow up period.

**Conclusions:**

Breastfeeding attitudes were encouraging in this population. Breastfeeding was almost universal. Use of infant formula was quite low, whereas cow or buffalo milk appeared to be popular supplementary foods.

## Background

Under-nutrition has a major role on child mortality and morbidity, posing a challenge to achieve the Millennium Development Goal 4 [1]. While suboptimal breastfeeding contributes to child under-nutrition [2], there are large variations in breastfeeding rates between countries [3]. In Nepal, breastfeeding is almost universal with reported rates of 98% and 70% for infants under six months being ever-breastfed and exclusively breastfed, respectively [4]. Breastfeeding attitudes of mothers and their intention to breastfeed are known to be associated with the duration of breastfeeding [5,6]. Such attitude and knowledge of breastfeeding can be affected by maternal, cultural and environmental factors [7-9].

Nepal is diversified in terms of ethnicity and ecology. The country has three ecological regions (Mountain, Hill and Plain) extending from east to west with 105 recorded ethnicities [4]. Existing information on infant feeding in Nepal has been largely derived from the Nepal Demographic and Health Surveys (DHS) [10]. As part of a large prospective cohort study in central Nepal, we report infant feeding information, attitudes and practices in this article.

## Methods

### Study design and location

This study was conducted in Kaski, a centrally located hills district of Nepal. The district has a 75% female literacy rate with 138,000 expected pregnancies per annum [11]. About half (49%) of the population belong to the upper caste of Indo-Aryan origin and about one-third (34%) belong to the Janajati caste who are mainly Tibeto-Burman people [11]. The rest (17%) are lower caste people also of Indo-Aryan origin.

This study is part of a large community-based prospective cohort study undertaken between December 2011 and November 2012 to investigate the utilisation of maternity services. Details about the sampling design and procedure have been described elsewhere [12,13]. Briefly, a cohort of 701 pregnant women of 5 months or more gestational age were recruited from the Kaski district and followed for six months after delivery.

### Data collection and ethics

The questionnaire used in the face-to-face interview was adapted from the validated Nepal Demographic and Health Survey and another breastfeeding instrument [14], and was subsequently pretested on 25 postpartum women for cultural appropriateness, content validity and understanding; see Additional file 1. Fifteen female data enumerators conducted the baseline interview from December 2011 to January 2012 to collect information on socio-demographics and obstetric characteristics of the pregnant women. The cohort of participants was then followed up at 4 weeks, 12 weeks and 22 weeks postpartum through repeated household visits by the same enumerators. Infant feeding information, including breastfeeding knowledge and support, husband’s and mother/mother-in-law’s preferences and feeding plans, was collected at 4 weeks during the second visit. Attitude towards breastfeeding was asked at the third visit, perception of sufficiency of breast milk was solicited at 4 and 22 weeks, whereas supplementary feeding was assessed at all three follow-up visits.

The study was approved by the Human Research Ethics Committee of Curtin University (approval number HR 130/2011), Ethical Review Board of the Nepal Health Research Council (approval number 88/2011) and the District Public Health Office of Kaski. An information sheet was distributed and read to each participant before obtaining her signed or thumb-print consent. Confidentiality of the information provided was maintained throughout the study.

### Statistical analysis

All data entry was performed by the first author, who further checked for correctness and consistency upon completion. Descriptive statistics were used to summarise the outcome variables and other variables of interest using the SPSS package version 20. Four levels of education were recorded: none, primary (1-5th grade), secondary (6-10th grade) and college (after 10th grade). Employment status was categorised as: employed (full-time salaried job), semi-employed (wage based labour, small business or employed abroad), and unemployed (agricultural, housewife or nothing). ‘Breastfeeding information’ was recorded as “none” if the woman did not receive such information from anywhere. Absence of ‘encourage to breastfeed’ meant there was no encouragement or motivation from anybody else to breastfeed after delivery. Similarly, lack of ‘advice on feeding method’ implied no one had advised the participant how to feed her newborn. Intended duration of breastfeeding and target time to introduce solid foods were initially recorded as continuous variables but recoded as categorical to facilitate analysis.

## Results

### Participant profile

Of the 639 women who took part in the second interview at 4 weeks after delivery, 615 and 515 participants were eventually followed up at the third (12 weeks) and fourth (22 weeks) interviews. Attrition was due to lost to follow up (n = 39) during the period and non-completers at 5 months (n = 85). Table 1 shows the socio-demographic characteristics of the cohort. At baseline about half the women were first time mothers, within the age range 20–24 years, and belonged to the upper caste. Almost 80% of them were unemployed while the majority (70.8%) received secondary or above education. These characteristics were similar at subsequent follow ups (Table 1). Moreover, no significant differences in these variables were found between drop outs and women remaining in the cohort at the two time points, suggesting that the loss-to-follow-up was random and no apparent bias was introduced through attrition.

**Table 1:** Characteristics of participants at 4, 12, and 22 weeks postpartum, Kaski District, Nepal

	**4 weeks n = 639**	**12 weeks n = 615**	**22 weeks n = 515**
**Characteristic**	**n (%)**	**n (%)**	**n (%)**
**Age (years)**			
15-19	91 (14.2)	91 (15.0)	75 (14.6)
20-24	324 (50.7)	313 (50.9)	264 (51.3)
25-40	224 (35.1)	211 (34.1)	176 (34.2)
**Parity**			
Primiparous	329 (51.5)	318 (51.7)	275 (53.4)
Multiparous	310 (48.5)	297 (48.3)	240 (46.6)
**Employment**			
Unemployed	509 (79.7)	495 (80.5)	408 (79.2)
Semi-employed	98 (15.3)	92 (15.0)	81 (15.7)
Employed	32 (5.0)	28 (4.6)	26 (5.0)
**Caste**			
Upper caste	339 (53.3)	327 (53.2)	268 (52.0)
Janajati	138 (21.7)	132 (21.5)	113 (21.9)
Lower caste	159 (25.0)	153 (24.9)	131 (99.4)
**Education**			
None	53 (8.3)	53 (8.6)	44 (8.5)
Primary	133 (20.8)	128 (20.8)	111 (21.6)
Secondary	238 (37.2)	234 (38.4)	195 (37.9)
College	215 (33.6)	200 (32.5)	165 (32.0)

### Infant feeding information and attitudes

As shown in Table 2, most of the mothers (73.6%) had received information on breastfeeding. Their main source of information was from health personnel (39.6%), followed by female community health volunteers (33.5%) and family members (24.8%). Often they had seen advertisements for infant formula (63.5%). Table 3 shows that 85% of mothers perceived their breast milk was sufficient at 4 weeks but the prevalence decreased significantly to 54.7% at 22 weeks postpartum (p < 0.001). In case of perceived insufficiency, about 80% of mothers at 4 weeks considered cow and/or buffalo milk as the main supplement, yet it reduced significantly to 63% at 22 weeks postpartum (p < 0.001).

**Table 2:** Infant feeding information and attitudes, Kaski District, Nepal (n = 639)

**Attitude**	**n**	**(%)**
Breastfeeding information*		
None	169	(26.4)
Health personnel	253	(39.6)
Female community health volunteers	214	(33.5)
Family members	158	(24.7)
Others (relatives, media, self-study, etc.)	13	(2.0)
**Intended breastfeeding duration**		
5-12 months	29	(4.5)
13-24 months	345	(54.0)
25-60 months	265	(41.5)
Mean (SD) months	28.6	(7.8)
**Target time to introduce solid foods**		
3-5 months	58	(9.1)
6 months	505	(79.0)
7-12 months	76	(11.9)
Mean (SD) months	6.1	(1.2)
**Decision on feeding method**		
Before becoming pregnant	95	(14.9)
Early in pregnancy	115	(18.0)
Late in pregnancy	76	(11.9)
After delivery	353	(55.2)
Advice on feeding method*		
None	206	(32.2)
Health personnel	279	(43.7)
Mother/mother-in-law	194	(30.4)
Husband	164	(25.7)
Encourage to breastfeed after delivery*		
None	123	(19.2)
Health personnel	336	(52.6)
Mother/mother-in-law	293	(45.9)
Husband	184	(28.8)
**Mother/mother-in-law’s preference**		
Prefers breastfeeding	504	(78.9)
Prefers bottle feeding	3	(0.5)
Do not care	17	(2.7)
Not discussed the matter	115	(18.0)
**Husband’s preference**		
Prefers breastfeeding	522	(81.8)
Prefers bottle feeding	4	(0.5)
Do not care	8	(1.3)
Not discussed the matter	105	(16.4)
**Attitude towards breastfeeding at 12 weeks (n = 615)**		
Totally not enjoyable	21	(3.3)
Not enjoyable	50	(7.8)
Neutral	132	(20.6)
Enjoyable	316	(49.4)
Very enjoyable	120	(18.8)

*Multiple responses.

**Table 3:** Perceived sufficiency of breastmilk at 4 and 22 weeks postpartum, Kaski District, Nepal

	**4 weeks n = 639**	**22 weeks n = 515**	**P**
**Perception**	**n (%)**	**n (%)**	
**Breastmilk sufficient**			
No	96 (15.0)	233 (45.3)	
Yes	543 (85.0)	282 ( (54.7)	<0.001
**Main alternative if insufficient**			
Cow/buffalo milk	508 (79.5)	325 (63.1)	<0.001
Infant formula	118 (18.5)	91 (17.7)	0.65
Porridge	13 (2.0)	99 (19.2)	<0.001

The average duration of intended breastfeeding was 28 months (range 5 to 60) and more than 95% of participants intended to breastfeed longer than 12 months. The average time planned to introduce solid foods was at 6 months (range 3 to 12) with 12% of women intending to do so after 6 months. About 55% of women decided what to feed their infant after giving birth. Although one-third of women (32.2%) reported that no one had advised them on feeding method, 43% said that they had been counselled by health personnel. In particular, about half the women (52.6%) were encouraged to breastfeed by health personnel after delivery. Moreover, approximately 80% of women perceived that their husband, mother or mother-in-law would prefer breastfeeding. Eleven percent of mothers reported that breastfeeding was not enjoyable at 12 weeks postpartum.

### Infant feeding

Table 4 presents the infant feeding practices by the Nepalese women. At birth, 581 of the 639 mothers fed colostrum as the newborn’s first feed, giving a breastfeeding rate of 90.9%. Almost all babies (99.3%) were breastfed at 4 weeks and the ‘any breastfeeding rate’ remained constant at 98% throughout the follow up until 22 weeks. Cow or buffalo milk was introduced to about a quarter (24.8%) and half (52.8%) of the infants at 12 weeks and 22 weeks, respectively, whereas infant formula was only fed as supplementary food to 6.3% and 13.4% of infants at the same time.

**Table 4:** Infant feeding practices, Kaski District, Nepal

	**At birth n = 639**	**4 weeks n = 639**	**12 weeks n = 615**	**22 weeks n = 515**
**Feeding method**	**n (%)**	**n (%)**	**n (%)**	**n (%)**
Breastfeeding	581 (90.9)	635 (99.3)	607 (98.6)	505 (98.0)
Cow/buffalo milk	4 (0.6)	92 (14.4)	153 (24.8)	272 (52.8)
Infant formula	43 (6.7)	11 (1.7)	39 (6.3)	69 (13.4)
Solids	0	0	23 (3.7)	156 (30.3)
Other fluids	11 (1.7)	0	0	0

## Discussion

This study confirmed the positive attitudes of women towards breastfeeding in central Nepal. They also appeared to receive good support and encouragement to breastfeed from health workers and family members. Indeed, birth preparedness programs have been implemented in the study district as part of the national mother and newborn survival strategy. This program incorporated lactation counselling sessions to pregnant women at both health facility and community levels. As observed in the present study, about one-third of women obtained such breastfeeding information from either female community health volunteers or health facility personnel, while about half of them received encouragement to breastfeed from health workers.

Breastfeeding is traditionally a popular practice in Nepalese society and mothers are committed to breastfeed for a long duration. This study found that almost all infants (98%) have been breastfed and their mothers intended to continue breastfeeding for an average of 28 months. Breastfeeding intention and social support have been found to be positively associated with breastfeeding duration [5]. In fact, the NDHS 2011 reported that half the children surveyed were breastfed up to 34 months [4]. A number of factors including unemployment and reliance on breast milk as the readily available infant food might have contributed to the continuing breastfeeding practice.

Many mothers perceived that breast milk was insufficient at 22 weeks and that cow or buffalo milk could be given as supplementary feeds. A cross-sectional study in urban Nepal reported that the main reason for introducing other foods before six months of age was perceived insufficient breast milk [15]. Another qualitative study of working mothers in peri-urban Kathmandu similarly concluded that mothers supplemented breastfeeding with infant formula and solid foods if they felt they did not have enough breast milk for their infants [16]. Unlike infant formula, cow and/or buffalo milk is locally available in both rural and urban areas. It is therefore not surprising to find that over half (52.8%) of the cohort of infants were given cow and/or buffalo milk at 22 weeks. The low usage of infant formula in urban areas of Nepal had been previously reported [17].

A major strength of this study was the prospective assessment of infant feeding attitudes from a large cohort of newly delivered women recruited from the community. Although maternal recall bias of infant feeding is expected to be minimal [18], generalizability of the findings to other parts of the country remains a limitation because the study was conducted in the central hills district of Nepal only. The Kaski district ranks third in terms of the human development index among 75 districts in Nepal with relatively high adult literacy rate (82% compared to 60.9% for Nepal overall), and *‘Gurung’* as the main Janajati people inhabiting this district [19]. Further qualitative studies are needed to explore the reasons behind their perceptions and attitudes on breastfeeding as well as the early introduction of supplementary feeds, together with quantitative analysis of the potential influencing factors, so that appropriate interventions can be developed to encourage breastfeeding for vulnerable population subgroups.

## Conclusions

Breastfeeding practices were encouraging in this population except the early introduction of supplementary feeds before 22 weeks. Average intention to breastfeed was 26 months. Use of infant formula was quite low, but supplementing breast milk with cow/buffalo milk was popular.

## Competing interests

The authors declare that they have no competing interests.

## Authors’ contributions

RK managed the project and coordinated data collection, performed data entry and drafted the manuscript. AHL contributed to data analysis and revision of the manuscript. VK assisted with data collection and project development. CWB developed the study protocol and revised the manuscript. All four authors read and approved the final version for publication.

## Supplementary Material

Additional file 1 Questionnaire: Infant feeding information and practices. Click here for file
