# Supplementary material for: Infant feeding information, attitudes and practices: a longitudinal survey in central Nepal
Source: Int Breastfeed J. 2014 Aug 28;9:14. doi: 10.1186/1746-4358-9-14 (PMC4149806; doi:10.1186/1746-4358-9-14)
Supplement: Additional file 1 — Questionnaire: Infant feeding information and practices. [file 1746-4358-9-14-S1.docx]

**Infant feeding information and practices (after delivery at 4 weeks)**

| When did you first decide how to feed your new baby? | 1. After my baby was born  2. Before I became pregnant  3. Early in my pregnancy  4. late in my pregnancy  5. Other…. |
| --- | --- |
| Who helped you decide whether you would bottle-feed or breast feed?  ***(Tick only one option)*** | 1. None  2. the baby’s father  3. Mother-in law  4. doctor/health worker  5. Other (specify)…….. |
| After delivery, did anyone support/encourage you to breastfeed?  ***(You may tick more than one option. Do not read the options. Tick options depending on what respondent says.)*** | 1. None supported  2. Doctor/HA  3. Nurses (ANM, SN)  4.My mother/mother-in-law  5. Friend/neighbour  6. Other (specify)……………….. |
| What was your baby’s first feed after he/she was born? | 1. Breast milk (or colostrum)  2. formula  3. cows’ or buffalos’ milk  4. Breast milk from another woman  5.Sugar water/ghee/honey  6. Plain water  7. Other (specify)……………. |
| How long after birth did you first breastfeed your baby? | 1. less than 1 hour  2. 1-4 hours  3. more than 4 hours, less than 1 day  4. More than 1 day after birth |
| How are you feeding your baby now?  ***(Tick only one option)*** | 1. Breastfeeding only  2. Bottle-feeding or cow/buffalo milk only  3. Mainly breast-feeding but also cow/buffalo milk  4. Mainly cow/buffalo milk but also breast-feeding  5. Other (specify)…………….. |
| Does your mother/mother-in law have any preferences for how you feed your baby? | 1. Yes, she prefers bottle-feeding  2. yes, she prefers breast-feeding  3. she doesn’t mind how I feed my baby  4. never really discussed the matter with her |
| Does the baby’s father have any preferences for how you feed your baby? | 1. Yes, he prefers bottle-feeding  2. yes, he prefers breast-feeding  3. she doesn’t mind how I feed my baby  4. never really discussed the matter with him |
| Have you received any breastfeeding education and information from anywhere on how to feed your baby? | 1. Yes  0. No |
| If yes, where did you receive the breastfeeding information? | ………………………………………….. |
| Is your breast milk enough for your baby? | 1. Yes  2. No  3. Do not know |
| If breast milk is not enough, what do you do? | ………….. |
| How long do you intend to breastfeed your baby? | ………………………months |
| At what age do you plan to start giving your baby its first solid food? | ……………………months |
| Have you seen any advertisements for infant formula? | 1. Yes  0. No |
| Have you experienced any of the following since you started breastfeeding?   - 1. A painful swelling of part of your breast   (pink, tender, hot, swollen area of the breast accompanied with fever or chills)   - 1. Cracked or sore nipples   2. Inverted nipples   3. Baby gets too much milk   4. Takes a long time before milk starts flowing at start of feed   5. Baby has problems sucking   6. Baby doesn’t wake up for feeds   7. Not enough milk or colostrum for baby   8. Other (specify) | 1. Yes 0. No  1. Yes 0. No  1. Yes 0. No  1. Yes 0. No  1. Yes 0. No  1. Yes 0. No  1. Yes 0. No  1. Yes 0. No |

**Follow-up for infant feeding practices at 12 and 22 weeks after delivery**

| What are you feeding your baby now?  ***(You may tick more than one options if applicable)*** | 1.Breastfeeding  2. Cow/Buffalo milk  3. Bottle-feeding  4. Porridge  5. Other (specify)……………… |
| --- | --- |
| Is your breast milk enough for your baby now? | 1. Yes  2. No  3. Do not know |
| If breast milk is not enough, how do you deal with it? | ……………………………….. |
| How enjoyable do you find breastfeeding? | 1.Totally not enjoyable  2. Not enjoyable  3. Neutral  4. Enjoyable  5. Very Enjoyable |
